# Protein Secondary Structure Prediction With a Reductive Deep Learning Method

**DOI:** 10.3389/fbioe.2021.687426

**Published:** 2021-06-15

**Authors:** Zhiliang Lyu, Zhijin Wang, Fangfang Luo, Jianwei Shuai, Yandong Huang

**Affiliations:** ^1^College of Computer Engineering, Jimei University, Xiamen, China; ^2^Department of Physics and Fujian Provincial Key Laboratory for Soft Functional Materials Research, Xiamen University, Xiamen, China; ^3^National Institute for Data Science in Health and Medicine, and State Key Laboratory of Cellular Stress Biology, Innovation Center for Cell Signaling Network, Xiamen University, Xiamen, China

**Keywords:** protein secondary structure, deep learning, multilayer perceptron, recurrent neural network, sequence profile

## Abstract

Protein secondary structures have been identified as the links in the physical processes of primary sequences, typically random coils, folding into functional tertiary structures that enable proteins to involve a variety of biological events in life science. Therefore, an efficient protein secondary structure predictor is of importance especially when the structure of an amino acid sequence fragment is not solved by high-resolution experiments, such as X-ray crystallography, cryo-electron microscopy, and nuclear magnetic resonance spectroscopy, which are usually time consuming and expensive. In this paper, a reductive deep learning model MLPRNN has been proposed to predict either 3-state or 8-state protein secondary structures. The prediction accuracy by the MLPRNN on the publicly available benchmark CB513 data set is comparable with those by other state-of-the-art models. More importantly, taking into account the reductive architecture, MLPRNN could be a baseline for future developments.

## 1. Introduction

Proteins are biomacromolecules that function in various life processes, many of which have been found as drug targets of human diseases (Huang et al., 2016; Li et al., 2021). The syntheses of proteins as long polypeptide chains or primary sequences take place in the ribosomes. Released from the ribosomes, the chains fold spontaneously to produce functional three-dimensional structures or tertiary structures (Anfinsen et al., 1961), which are usually determined by experiments, including X-ray crystallography, cryo-electron microscopy, and nuclear magnetic resonance spectroscopy. However, these experiments are often time consuming and expensive, which to a large extent explains the gap between the number of protein structures (~150,000) deposited in the Protein Data Bank (PDB) (Berman et al., 2002) and that of sequences (~140,000,000) stored in the UniProtKB/TrEMBL database (The UniProt Consortium, 2017, 2018). Therefore, it is of importance to develop efficient computational methods for protein structure prediction. The three-dimensional structure of a protein is determined most by its amino acid sequence (Baker and Sali, 2001), indicating the possibility of theoretical prediction of a protein structure from its amino acid sequence.

Protein secondary structures are characterized as local structures that are stabilized by hydrogen bonds on the backbone and considered as the linkages between primary sequences and tertiary structures (Myers and Oas, 2001; Zhang, 2008; Källberg et al., 2012). According to the distinct hydrogen bonding modes, generally three types of secondary structures have been identified, namely helix (H), strand (E), and coil (C), where the helix and strand structures are most common in nature (Pauling et al., 1951). Later in 1983, a finer characterization of secondary structures was proposed. In the new classification calculated by DSSP algorithm, previous 3 states are extended to 8 states, including α-helix (H), 3_10_ helix (G), π-helix (I), β-strand (E), β-bridge (B), β-turn (T), bend (S), and loop or others (C) (Kabsch and Sander, 1983), among which the α-helix and β-strand are the principal structure features.

The 3-state or Q3 prediction problem has been extensively studied since 1974 (Chou and Fasman, 1974). As summarized by Stapor and coworkers, the computational models reported after 2007 can provide the prediction accuracy of 80% and above (Smolarczyk et al., 2020). Until 2018, the theoretical limit 88% of the Q3 protein secondary structure prediction was achieved first by Lu group (Zhang et al., 2018). At the same time, it is noticed that the 8-state or Q8 prediction would provide more valuable information. For instance, π-helix is found abundant and associated with activities in some special proteins (Cooley et al., 2010). As a result, over the few years many efforts have been made, trying to solve the Q8 prediction problem, which is much more complicated and challenging (Li and Yu, 2016; Wang et al., 2016; Fang et al., 2017; Heffernan et al., 2017; Zhang et al., 2018; Krieger and Kececioglu, 2020; Uddin et al., 2020; Guo et al., 2021) If not otherwise specified, the models discussed in this paper are non-template based. The Q8 prediction accuracy has reached 70% and at present the best record is 77.73% (Uddin et al., 2020). Thus, there is still a deviation of about 10% from the theoretical limit of 88% (Rost et al., 1994).

Over the past few decades, a variety of state-of-the-art methods have been developed to improve Q3 or Q8 prediction accuracy and most progresses are contributed by machine learning based models (Li and Yu, 2016; Wang et al., 2016; Fang et al., 2017; Heffernan et al., 2017; Zhang et al., 2018; Krieger and Kececioglu, 2020; Uddin et al., 2020; Guo et al., 2021) So far as we know, the predictive power of a machine learning model is governed mainly by two elements, namely feature representation and algorithm. For instance, the introduction of sequence evolutionary profiles from multiple-sequence alignment (Rost and Sander, 1993), such as position-specific scoring matrices (PSSM) (Jones, 1999), improves prediction accuracy significantly (Zhou and Troyanskaya, 2014). In addition to PSSM, either the hidden Markov model (HMM) profile (Guo et al., 2021) or amino acid parameters (Zhang et al., 2018) can also contribute to the improvement of prediction accuracy. As to a machine learning algorithm, the major task is to capture either local or non-local dependencies from the input features using different neural network architectures. For instance, a specific neural network, namely convolutional neural network (CNN) (LeCun et al., 1998), is successful in capturing short-range features. At the same time, the recurrent neural network (RNN) equipped with bidirectional gate current unit (BGRU) (Cho et al., 2014) or long short-term memory (LSTM) (Hochreiter and Schmidhuber, 1997) can be used to capture long-range dependencies. CNN and RNN architectures were integrated for the first time in the DCRNN model to predict protein secondary structures (Li and Yu, 2016; Zhang et al., 2018). Some models employ different deep learning architectures, such as the deep conditioned neural field (DeepCNF) (Wang et al., 2016) and the deep inception-inside-inception network (Deep3I) (Fang et al., 2017; Uddin et al., 2020). In particular, the model SAINT that incorporates self-attention mechanism and Deep3I provides up-to-date the best Q8 prediction accuracy (Uddin et al., 2020).

Noting that as the neural network architecture gets more complex or deeper, the number of parameters grows. In this work, a reductive neural network architecture MLPRNN has been proposed that include a two-layer stacked bidirectional gated recurrent unit (BGRU) block capped by two multilayer perceptrons (MLP) at both sides, like a sandwich. Encouragingly, the prediction accuracy for Q3 and Q8 reach 83.32 and 70.59%, respectively, comparable with other state-of-the-art methods developed recently. More importantly, taking into account the reductive architecture, MLPRNN would provide an extensible framework for future developments.

## 2. Methods and Materials

### 2.1. Data Sets

In this work, two publicly available data sets, CB6133-filtered and CB513 (Zhou and Troyanskaya, 2014), which have been widely applied in protein secondary structure prediction (Li and Yu, 2016; Fang et al., 2017; Zhang et al., 2018; Guo et al., 2021), were used to train and test the new model, respectively. The CB6133-filtered is the result of removing the sequences that have >25% identity with the CB513 and the redundancy with the CB513 from the original CB6133. As expected, the distributions of 8 states with respect to the CB6133-filtered and CB513 are similar (Supplementary Figure 6).

#### 2.1.1. CB6133-Filtered

An open-source protein sequence data set, namely CB6133-filtered, was employed for training in this work (Zhou and Troyanskaya, 2014). CB6133-filtered is a large non-homologous sequence and structure data set that contains 5,600 training sequences. This data set was produced with the PISCES Cull PDB server, a public server for culling sets of protein sequences from the Protein Data Bank (PDB) by the sequence identity and structural quality criteria (Wang and Dunbrack, 2003). Notably, the data set was created with better than 2.5Å resolution while sharing less than 30% identity.

#### 2.1.2. CB513

The testing data set CB513 was introduced by Cuff and Barton (Cuff and Barton, 1999, 2000). Noting that the length of one sequence is longer than the maximal of 700, this sequence has been split into two overlapping sequences. As a result, CB513 contains 514 sequences. Both CB6133-filtered and CB513 data sets can be downloaded via Zhou's website.

### 2.2. Input Features

#### 2.2.1. PSSM Profile

Statistically, homologous proteins often have similar secondary structures. Thus, all homologous proteins can be grouped into a family through the multiple sequence alignment (MSA) with a fitting cutoff (Sander and Schneider, 1991). Then the approximate structure of the family can be predicted. Apparently, the MSA gives much more structural information than one single sequence (Rost and Sander, 1993). One of the most popular position-specific profile of proteins is the PSSM (Jones, 1999), which can be produced by the PSI-BLAST algorithm (Altschul et al., 1997). The PSSM dimension of a sequence is *N*×*S*, where *N* and *S* denote the types of amino acids and the length of the sequence, respectively. Normally, N is 20 that corresponds to the 20 standard amino acid types. Here, one additional type, marked as X, was added to the PSSM profile to represent non-standard amino acids. Thus, N is 21 instead of 20 for the PSSM profile. According to the PSI-BLAST, each position of amino acids gets a score of hit that denotes the appropriate probability of the amino acid staying in this position solidly. For instance, if the score of the hit is high, a position is supposed to be conserved. Otherwise, the position is not likely a conserved site (Gribskov et al., 1987; Jeong et al., 2010). Usually, a sigmoid function is applied to restrain the scores of the hits that range from 0 to 1 (Jones, 1999).

#### 2.2.2. HMM Profile

Recently, it has been demonstrated that the combination of HMM and PSSM profiles as input of the model DNSS2 can improve the Q8 prediction accuracy by about 2% (Guo et al., 2021). Thus, in this work, we follow the scheme above and the PSSM and HMM profiles were used as input. The HMM profile was calculated with the HHblits (Remmert et al., 2012), a software that can convert amino acid sequences into hidden Markov model profiles by searching specific databases iteratively. The database used in this work is the publicly available *uniclust30_2016_03.tgz*. The columns in the HMM profile correspond to the 20 amino acid types. In each column, a substitution probability is provided based on its position along the protein sequence (Smolarczyk et al., 2020). Finally, the values generated by the HHblits were transformed to the linear probabilities, which can be formulated as follows:

(1) $$\begin{array}{l} p=2^{-N/1000} \end{array}$$

where N denotes the score number from the profile (Sharma et al., 2016). Compared to the sequence-search tool PSI -BLAST, HHblits is faster because of its discretized-profile prefilter. Also, HHBlits is more sensitive than PSI-BLAST (Remmert et al., 2012).

### 2.3. Model Design

The reductive model MLPRNN proposed in this study is composed by one BGRU and two MLP blocks. In this section, MLP and BGRU will be introduced separately. Followed is the explanation in details of the overall architecture.

#### 2.3.1. MLP

**Figure 1:**
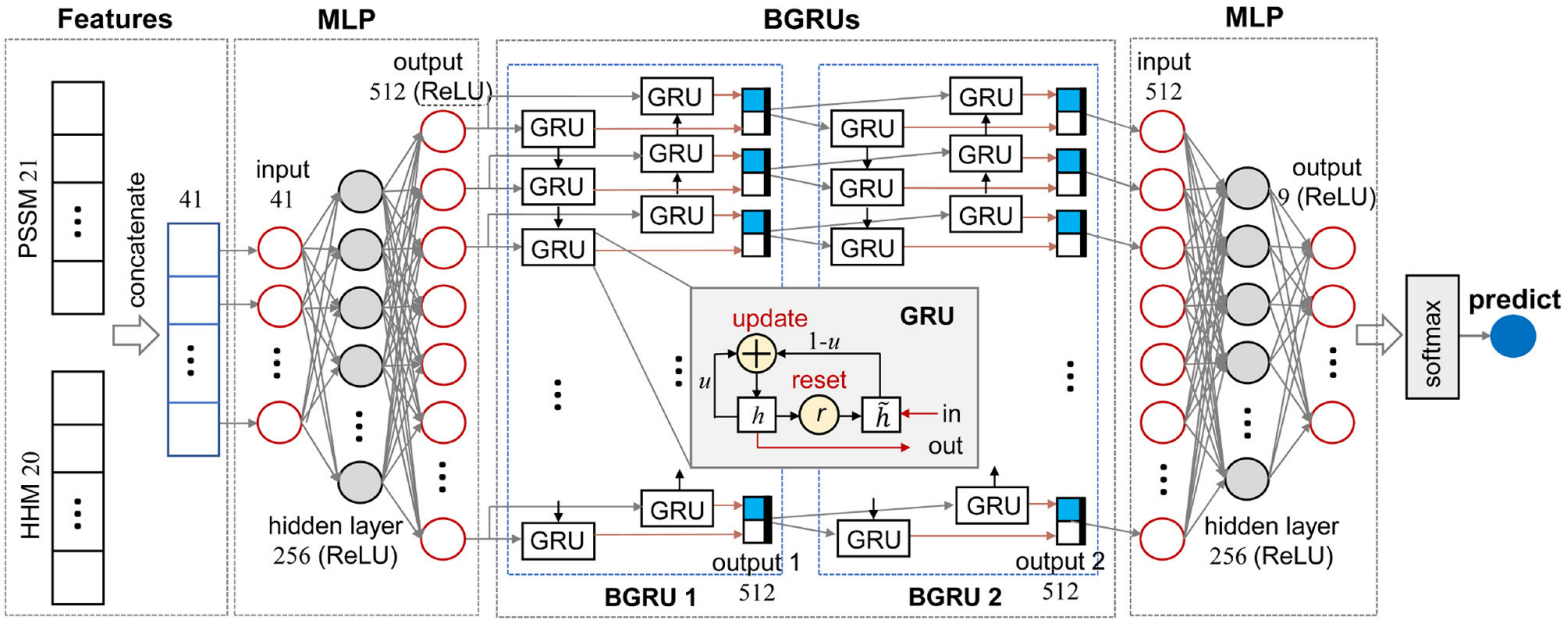
Schematic diagram of the MLPRNN model.

The multi-layer perceptron (MLP) is a reductive neural network with at least three layers, namely an input layer, a hidden layer, and an output layer. Taking the three-layer MLP exploited in this study as an example, as illustrated in Figure 1, each neuron at the hidden layer integrates the messages from all input nodes and spreads the integrated message to all neurons at the output layer. A linear function is used to adjust the number of neurons at each layer. Each neuron need to work with a non-linear activation function, such as Rectified Linear Unit (ReLU), and a dropout method.

#### 2.3.2. BGRU

In this study, the bidirectional gate current units (BGRUs) were used to capture long-range dependencies in the amino acid sequences. Assuming the number of hidden units is k and the input of a GRU(t) is (*l*_*t*_, *h*_*t*−1_). The activated reset gate *r*_*t*_, update gate *u*_*t*_, internal memory cell ${\tilde{h}}_{t}$, and GRU output $h_{t}\left(\in {\mathbb{R}}^{k}\right)$ can be expressed as follows:

(2) $$\begin{array}{l} r_{t}=\sigma \left(W_{lr}l_{t}+W_{hr}h_{t-1}+b_{r}\right) \end{array}$$

(3) $$\begin{array}{l} u_{t}=\sigma \left(W_{lu}l_{t}+W_{hu}h_{t-1}+b_{u}\right) \end{array}$$

(4) $$\begin{array}{l} {\tilde{h}}_{t}=tanh\left(W_{l\tilde{h}}l_{t}+W_{h\tilde{h}}\left(r_{t}⊙h_{t-1}+b_{\tilde{h}}\right)\right) \end{array}$$

(5) $$\begin{array}{l} h_{t}=u_{t}⊙h_{t-1}+\left(1-u_{t}\right)⊙{\tilde{h}}_{t} \end{array}$$

where *W*_*lr*_, *W*_*hr*_, *W*_*lu*_, *W*_*hu*_, $W_{l\tilde{h}}$, and $W_{h\tilde{h}}$ (∈ ℝ^3*q*×*k*^) denote weight matrices. *b*_*r*_, *b*_*u*_, and $b_{\tilde{h}}$ (∈ ℝ^*k*^) are bias terms. ⊙, σ, and *tanh* stand for element-wise multiplication, sigmoid, and hyperbolic functions, respectively (Li and Yu, 2016). As illustrated in the inset of Figure 1, each GRU contains one input and one output. A BGRU layer, such as BGRU 1 in Figure 1, not only learns input features from head to tail, but also tail to head, so as to catch the dependencies at both sides. Thus, a BGRU need read input features twice. In the end, outputs of two GRU chains are merged together as the final output.

#### 2.3.3. Overview of MLPRNN

Figure 1 illustrates the data stream of an amino acid in the sequences and the other dimension perpendicular to the plot is the amino acid sequences. As illustrated in Figure 1, MLPRNN has a sandwich like architecture where a two-layer stacked BGRU block is capped by two MLP blocks at both sides. Both MLP blocks have one hidden layer. In specific, 41-dimensional features are taken as the input of the first MLP block. The dimensions of the input, hidden, and output layers in the first MLP block are 41, 256, and 512, respectively. The BGRU block is fed with the 512-dimensional output of the first MLP. The BGRU block is followed by the other MLP block with one hidden layer too. The dimensions of the input, hidden, and output layers are 512, 256, and 9, respectively. Finally, the prediction is made by a softmax unit fed by the output of the second MLP block. The dimensions of the hidden and output layers in the MLP blocks are selected based on the prediction accuracy. As shown in Supplementary Table 1, the combination of the dimensions 256 and 512 give not only the best Q8 prediction, but also the fastest convergence. From Supplementary Table 2, one can see that the model with two-layer stacked BGRU block gives best performance in view of accuracy as well as efficiency. For instance, the models with respect to two-layer and three-layer stacked BGRU blocks give similar accuracies, but the former has less parameters. Thus, the two-layer stacked BGRU block is chosen in this study.

### 2.4. Implementation Details

In all experiments, the optimizer named Adam was used during the training to calculate and update the parameters of the model. The default original learning rate is set 0.001, which decreases every 10 epochs with the rate of 0.997. All sequences were padded with zero if the sequence length is shorter than 700. As a consequence, zero could be learned by the model, which is undesired. To remove the effect of the zero class, the Multiple Cross-Entropy Loss function was employed, which is based on the cross-entropy loss function. The weight constraint of dropout with the parameter *p* = 5 was applied to avoiding over fitting by BGRUs and the tails of MLPs. Our experiments were implemented under the PyTorch (version 1.7.1) environment and the model was trained on a single NVIDIA Titan RTX GPU with 24 Gigabyte (GB) memory. Each experiment in this work was trained and tested for at least 3 times and the best result was taken as the final solution. In this work, the average of the loss over the last 10 epochs was used to determine at which epoch the convergence was reached for the testing set.

### 2.5. Performance Evaluation

The Q Score formulated as Equation (6) has been widely used to examine protein secondary structure predictions. In brief, it measures the percentage of residues for which the predicted secondary structures are correct (Wang et al., 2016).

(6) $$\begin{array}{l} Q_{m}=100\%\times \frac{\overset{m}{\underset{i=1}{\sum }}N_{corr}\left(i\right)}{N} \end{array}$$

where *m* indicates the number of classes. *m* = 3 and *m* = 8 correspond to Q3 and Q8 predictions, respectively (Lee, 2006). *N*_*corr*_(*i*) is the number of correctly predicted residues for state i and *N* is the total number of residues.

## 3. Results and Discussion

### 3.1. Prediction Accuracy

Q3 and Q8 prediction accuracy have been estimated by the proposed model MLPRNN and compared with the values by another 5 state-of-the-art methods that also used CB513 for testing. Here Q8 is transformed to Q3 by treating 3_10_-helix and π-helix as α-helix (H) and merging β-bridge (B) to β-strand (E). As to the rest, turn (T) and bend (S) are treated as coil (C). As illustrated in Table 1, the prediction accuracy for either Q3 or Q8 by MLPRNN is at the same level with other state-of-the-art methods. In particular, the Q8 prediction accuracy obtained by the new model is about 1 and 3% lower than those given by CRRNN (Zhang et al., 2018) and DNSS2 (Guo et al., 2021), respectively. Here, the DNSS2 integrates 6 deep learning architectures, which is much more complex than the present MLPRNN. In addition to the PSSM and HMM profiles, another three input features were utilized in the DNSS2 model (Guo et al., 2021). With respect to CRRNN, the training set TR12148 applied by this model is about twice larger than the CB6133-filtered used in this work (Zhang et al., 2018). Thus, the present MLPRNN could be improved with more input features such as the ones introduced by DNSS2 or a larger training dataset like the TR12148. It should be noted that MLPRNN and DNSS2 share the same method of mapping Q8 to Q3. Although CRRNN and DeepCNF use another method for the transformation. In specific, α-helix (H), β-strand (E), and the rest 6 states in Q8 form the 3 classes of Q3, respectively. It has been reported that the selection of the transformation method from Q8 to Q3 can influence prediction performance to some extent (Cuff and Barton, 1999). Indeed, replacing the present method of converting Q8 to Q3 with the one employed by CRRNN, the prediction accuracy of Q3 by MLPRNN increases from 83.32 to 85.38%, slightly higher than 85.30% by CRRNN.

**Table 1:** Q3 and Q8 prediction accuracy (%) comparison.

**Method**	**References**	**Q3**	**Q8**
DeepCNF	Wang et al., 2016	82.30	68.30
MUFOLD-SS	Fang et al., 2017	82.98	71.05
BGRUCB	Drori et al., 2018	82.85	70.10
CRRNN	Zhang et al., 2018	85.30	71.40
DNSS2	Guo et al., 2021	82.56	73.36
MLPRNN		83.32	70.59

### 3.2. Convergence Rate

**Figure 2:**
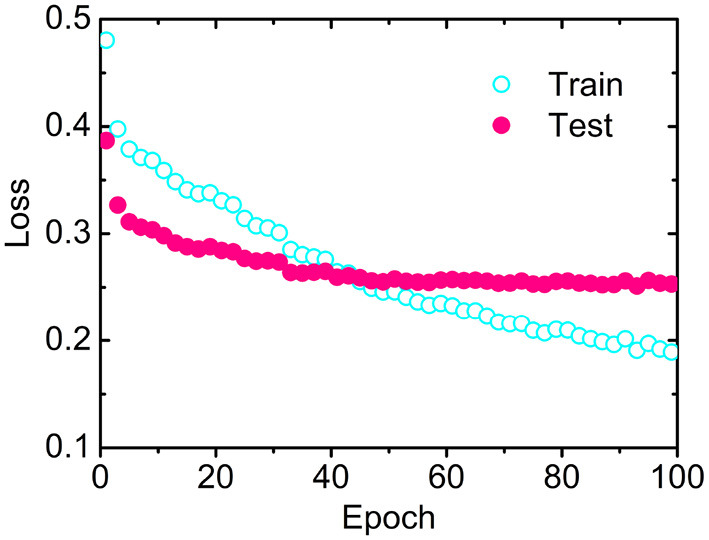
Losses as a function of epoch by MLPRNN for the training (open circles) and testing (solid circles) data sets, respectively.

The losses as a function of epoch for the training (CB6133-filtered) and testing (CB513) data sets, respectively, have been calculated to examine the convergence. As illustrated in Figure 2, the loss for CB513 drops from 0.39 to 0.30 within 6 epochs and stabilized or converged around 0.26 for another 38 epochs. The following two experiments have been designed, trying to explain the fast convergence of loss for CB513 by MLPRNN. First, the MLP blocks were removed from MLPRNN. As a result, the number of epochs required for loss convergence increases to 70 (Supplementary Figure 1), which is expected as BGRU is known as slow in learning when compared with other neural network architectures (Bradbury et al., 2016). Next, MLP was replaced with CNN, and the resulting convergence rate is similar with that by the original MLPRNN (see Supplementary Figures 2, 3). Thus, the sandwich-like reductive architecture itself is responsible for the fast loss convergence. It should be noted that MLP is more suitable than CNN for this model in terms of prediction accuracy, which will be discussed later.

### 3.3. Feature Analysis

Feature representation is essential for the prediction of protein secondary structures. In this work, the input features are represented by the concatenation of PSSM and HMM profiles, both of which transfer the evolutionary information for amino acids in the sequences. Thus, it is of interest to examine the impacts of the two profiles separately. The loss convergence plots of the two experiments can be found in Supplementary Figures 4, 5. From Table 2, one can see that the prediction accuracy with PSSM profile is higher than that with HMM profile. In particular, the discrepancy is about 7% for Q8 prediction. However, when PSSM is combined with HMM, the prediction accuracy is improved by about 1% for both Q3 and Q8 predictions, implying that HMM profile is complementary to PSSM profile, which is consistent with the result obtained by the DNSS2 model (Guo et al., 2021).

**Table 2:** Q8 prediction accuracy (%) with different input features.

**Model**	**Q3**	**Q8**
PSSM	82.27	69.50
HMM	80.51	62.49
PSSM+HMM	83.32	70.59

Noting that the PSSM profile was generated by the PSI-BLAST, a profile-sequence alignment method, and the HMM profile was generated by the method HHblits that uses both profile-sequence alignment and profile–profile alignment. It has been suggested that the HHblits method is more sensitive to identify distant homologous sequences than the PSI-BLAST, indicating different sensitivity and specificity between the two methods (Guo et al., 2021), which might explain the distinct performances between PSSM and HMM profiles found in the current protein secondary structure prediction. In specific, the PSI-BLAST method is perhaps more sensitive to the sequence homology of the datasets utilized in this work. In addition, the present HMM profile was generated based on a smaller sequence database, which might influence the accuracy of the HMM profile and the resulting prediction accuracy.

### 3.4. Model Analysis

The current reductive model MLPRNN is constructed by only a two-layer stacked BGRU block capped by two MLP blocks, facilitating detailed model analysis. To examine the impact of adding MLP blocks to both sides of BGRU block, the input data were trained with BGRU block alone and the resulting prediction accuracies are 73.22 and 61.95% for Q3 and Q8, respectively, about 10% lower than those by the original MLPRNN where MLP blocks are present. Apparently, the MLP blocks in the MLPRNN model are essential to the prediction.

**Figure 3:**
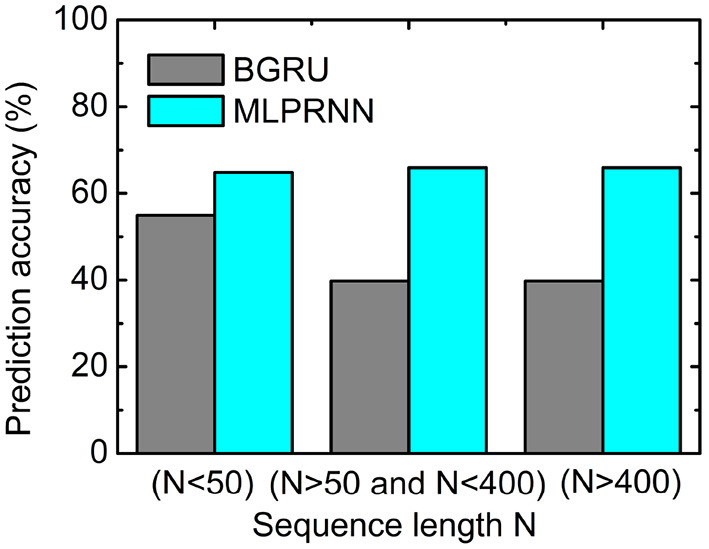
Prediction accuracy obtained by the multilayer perceptron (MLP)-removed MLPRNN model (gray) and the original MLPRNN model (cyan) for three sequence length regions.

Further, to investigate where the MLP-related improvement occurs, the sequences for testing were split into three groups according to the length N of a sequence. As illustrated in Figure 3, the prediction accuracy where N is larger than 50 is below 40%, about 15% lower than that where N is smaller than 50. When the MLP blocks are added, the prediction accuracies are all above 60% for the three length regions, indicating that MLP blocks could help capture very long-range dependencies. The experiment above highlights that the two MLP blocks are indispensable complementary to the BGRU block for protein secondary structure prediction.

CNNs have been used to couple with BGRUs for protein secondary structure prediction since 2016 (Li and Yu, 2016; Zhang et al., 2018). Therefore, it is of interest to see if the current framework works with CNNs too. In this experiment, MLPs in the MLPRNN model were replaced by CNNs where the kernel size *k* equals 3 or 7. Noting that a CNN with the kernel size *k* = 1 is equivalent to a MLP, MLPRNN is renamed as CNN(k = 1)BGRU in Table 3. From Table 3, one can see that the prediction accuracy reduces as the kernel size increases, which is more evident for Q8 prediction, demonstrating that MLPs match better with BGRUs than CNNs under the proposed reductive architecture.

**Table 3:** Q3 and Q8 prediction accuracy (%) where multilayer perceptrons (MLPs) in the MLPRNN are replaced by convolutional neural networks (CNNs).

**Model**	**Q3**	**Q8**
CNN (k = 1) BGRU	83.32	70.59
CNN (k = 3) BGRU	82.89	68.30
CNN (k = 7) BGRU	82.14	67.46

Standard RNNs include LSTMs and GRUs. Thus, it is worth investigating the effect of replacing BGRUs with bidirectional LSTMs (BLSTMs). As presented in Supplementary Table 2, the BLSTMs show no impact on the prediction accuracy except for the reduced convergence rate, which is mainly due to the increased amount of parameters.

### 3.5. Prediction Accuracy for Individual Q8 States

Apart from the overall accuracy, the predictive precision for each class of Q8 would provide more useful information. Thus, the prediction accuracies for all Q8 states were calculated and listed in Table 4 that includes the results by the MLPRNN model and the experiments mentioned above. Here, the labels are ordered based on the counts of 8 states in the training data set. It is evident that the prediction of T by BGRU is poor when compared with those by others, indicating that MLP or CNN blocks in the current framework are essential to predict the turn structure. Interestingly, only the MLPRNN model fed with at least PSSM profile is able to distinguish S or G from other states, though the prediction accuracy is still low.

**Table 4:** Prediction accuracy (%) for Q8 states.

**Label**	**Types**	**Count**	**BGRU^**a**^**	**MLPRNN**	**MLPRNN**	**MLPRNN**	**CNN(k = 3)**	**CNN(k = 7)**
					**(PSSM)^**b**^**	**(HMM)^**c**^**	**BGRU^**d**^**	**BGRU^**e**^**
H	α-helix	405560	91.28	92.42	92.32	90.72	93.15	92.88
E	β-strand	255887	81.52	83.34	81.67	82.04	84.20	82.28
L	Coil	225493	64.48	68.34	64.97	67.22	71.22	71.36
T	Turn	132980	17.88	54.02	50.78	46.55	55.92	52.73
S	Bend	97298	6.73	26.83	27.91	0	0	0
G	3_10_-helix	46019	1.50	25.73	29.92	0	0	0
B	β-bridge	12096	0	0	0	0	0	0
I	π-helix	209	0	0	0	0	0	0

a *MLPs are removed.*

b *Input features are represented by PSSM profile.*

c *Input features are represented by HMM profile.*

d *MLPs are replaced by CNNs with the kernel size k = 3.*

e *MLPs are replaced by CNNs with the kernel size k = 7*.

From the third column of Table 4, one can see that the count of S or G type is much smaller than those with respect to the four most populated types, namely H, E, L, and T. Under such a limited number of samples, accurate feature extraction is essential for the prediction of S or G type. When CNNs are used, local features are extracted preliminarily at the convolution step before entering the neural network. Here, the range of the local features is determined by the kernel size. When the kernel size of 3 or above is used, some very local information, which are critical for the prediction of S or G type, could be missed during the convolution step. As a consequence, the following training in the neural network would be affected. In that case, the kernel size of 1, which is equivalent to MLP employed by the proposed MLPRNN, might be necessary.

From the prediction accuracies for individual Q8 states, it is found that HMM profile compensates PSSM profile by improving the prediction accuracies of H, E, L, and T types. Adding HMM profile to PSSM profile as input, however, reduces the prediction accuracies of the two less populated states, namely S and G. In association with the discussion on input features above, the poor prediction of either G or S type with the HMM profile alone as input might be due to the underlying effect of sequence homology.

The results above have provided two messages, which might be useful for future development. First, PSSM profile is better than HMM profile in representing bend and 3_10_-helix states. Second, MLP is more suitable than CNN in predicting the two states.

## 4. Conclusion

In this study, we proposed a reductive deep-learning architecture MLPRNN for protein secondary structure prediction. Based on the benchmark CB513 data set, the prediction accuracy for either Q3 or Q8 by MLPRNN is comparable with those by other state-of-the-art methods, verifying the validity of this reductive model. From the comparative experiments, it is found that MLPs are non-trivial to the proposed model. First, MLPs contribute a lot to secondary structure prediction made by MPLRNN, especially at the long sequence length side. Besides, the reductive model performs better in the presence of MLPs instead of CNNs. The impact of input features have been studied too. It is revealed that, in contrast to PSSM profile, HMM profile fails in representing two less populated states, bend and 3_10_-helix. In addition, the prediction of the two states fails too if the MLPs in the MLPRNN model are replaced with CNNs. Encouragingly, the original MLPRNN model in the presence of MLPs could capture features of the two states represented by PSSM profile. Finally, the MLPRNN model proposed in this study has provided a reductive and extensible deep learning framework, facilitating the incorporation of more sophisticated algorithms or new features in future for further improvement.

## Data Availability Statement

The code of MLPRNN and the relevant data can be downloaded from https://gitlab.com/yandonghuang/mlpbgru.

## Author Contributions

YH and ZW conceived the idea of this research. ZL and ZW performed the model implementation. ZL performed the data collection, training, and testing. ZL and FL performed the data analysis. YH, ZL, and JS wrote the manuscript. YH and JS supervised the research and reviewed the manuscript. All authors contributed to the article and approved the submitted version.

## Conflict of Interest

The authors declare that the research was conducted in the absence of any commercial or financial relationships that could be construed as a potential conflict of interest.
