# Supplementary material for: Protein Secondary Structure Prediction With a Reductive Deep Learning Method
Source: Front Bioeng Biotechnol. 2021 Jun 15;9:687426. doi: 10.3389/fbioe.2021.687426 (PMC8240957; doi:10.3389/fbioe.2021.687426)
Supplement: Supplementary file 1 [file Data_Sheet_1.PDF]

Supplementary Material

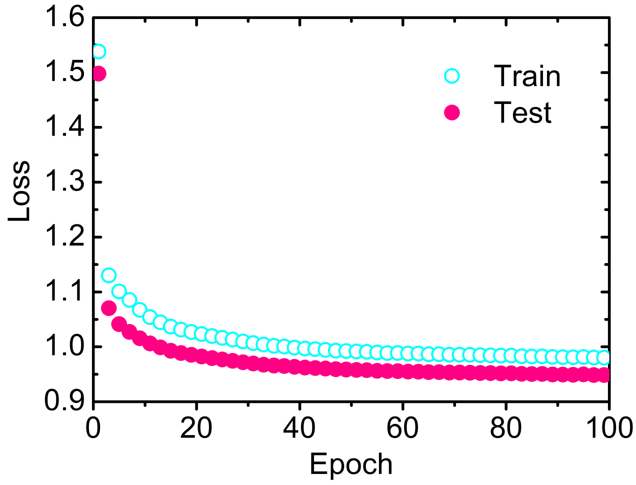

Figure S1. Losses as a function of epoch for the training CB6133-filtered (open circles) and testing CB513 (solid circles) data sets respectively where MLPs are removed.

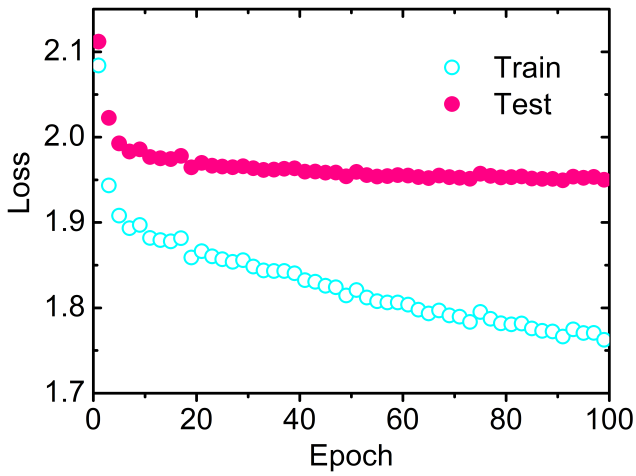

Figure S2. Losses as a function of epoch for the training CB6133-filtered (open circles) and testing CB513 (solid circles) data sets respectively where MLPs are replaced by CNNs with the kernel size k of 3.

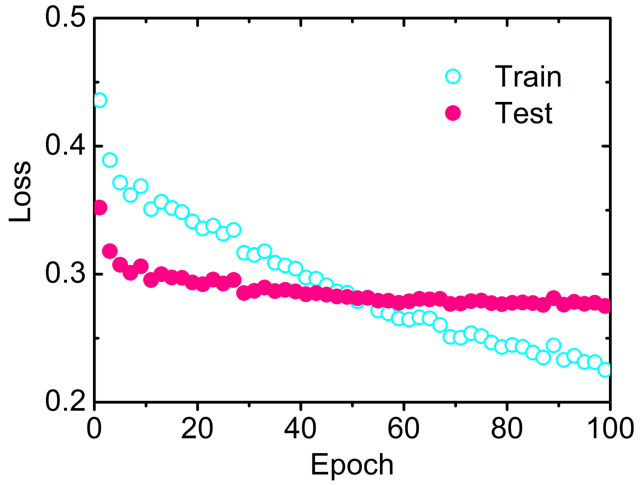

Figure S3. Losses as a function of epoch for the training CB6133-filtered (open circles) and testing CB513 (solid circles) data sets respectively where MLPs are replaced by CNNs with the kernel size k of 7.

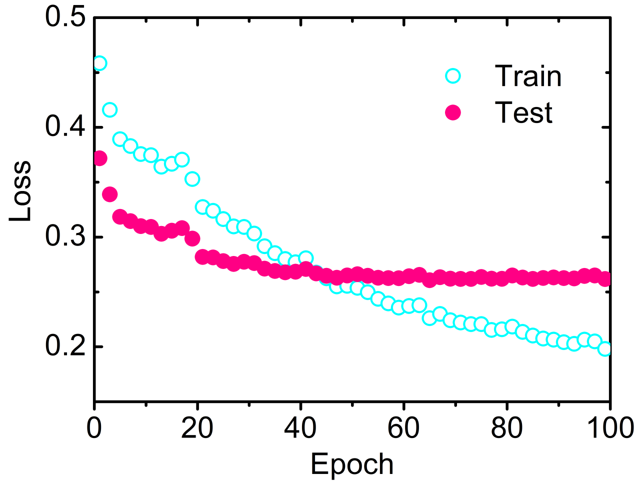

Figure S4. Losses as a function of epoch for the training CB6133-filtered (open circles) and testing CB513 (solid circles) data sets respectively where the input features are represented with PSSM profile.

Table S1. Predictive performances with different dimensions of the hidden and output layers in the first MLP block.

Input	Hidden	Output	Accuracy	Epoch
41	64	128	68.5	73
41	128	256	69.7	48
41	256	512	70.6	44
41	512	1024	69.3	90

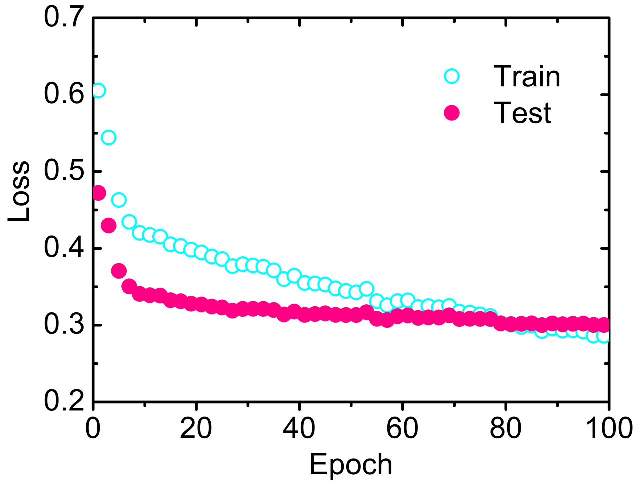

Figure S5. Losses as a function of epoch for the training CB6133-filtered (open circles) and testing CB513 (solid circles) data sets respectively where the input features are represented with HMM profile.

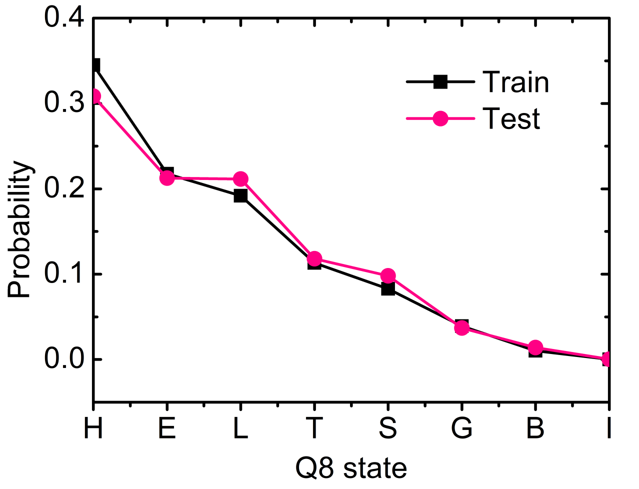

Figure S6. The probabilities of 8 states in the training CB6133-filtered (open circles) and testing CB513 (solid circles) data sets respectively.

Table S2. Predictive performances of varying the number of BGRU layer or replacing BGRU with BLSTM.

Model	Layer	Accuracy	Epoch	Parameter
BGRU	1	69.31	36	1458697
BGRU	2	70.59	44	2641417
BGRU	3	70.61	47	3824137
BLSTM	2	70.30	58	3429897
